# Systematic analysis of MADS-box gene family in the U’s triangle species and targeted mutagenesis of *BnaAG* homologs to explore its role in floral organ identity in *Brassica napus*

**DOI:** 10.3389/fpls.2022.1115513

**Published:** 2023-01-12

**Authors:** Min Song, Yanfeng Zhang, Qingli Jia, Shuhua Huang, Ran An, Nana Chen, Yantao Zhu, Jianxin Mu, Shengwu Hu

**Affiliations:** ^1^ State Key Laboratory of Crop Stress Biology in Arid Areas and College of Agronomy, Northwest Agriculture and Forestry University, Yangling, Shaanxi, China; ^2^ Hybrid Rapeseed Research Center of Shaanxi Province, Yangling, Shaanxi, China

**Keywords:** Brassica, U’s triangle, MADS-box genes, floral organ development, AGAMOUS

## Abstract

MADS-box transcription factors play an important role in regulating floral organ development and participate in environmental responses. To date, the MADS-box gene family has been widely identified in *Brassica rapa* (*B. rapa*), *Brassica oleracea* (*B. oleracea*), and *Brassica napus* (*B. napus*); however, there are no analogous reports in *Brassica nigra* (*B. nigra*), *Brassica juncea* (*B. juncea*), and *Brassica carinata* (*B. carinata*). In this study, a whole-genome survey of the MADS-box gene family was performed for the first time in the triangle of U species, and a total of 1430 MADS-box genes were identified. Based on the phylogenetic relationship and classification of MADS-box genes in *Arabidopsis thaliana* (*A. thaliana*), 1430 MADS-box genes were categorized as M-type subfamily (627 genes), further divided into Mα, Mβ, Mγ, and Mδ subclades, and MIKC-type subfamily (803 genes), further classified into 35 subclades. Gene structure and conserved protein motifs of MIKC-type MADS-box exhibit diversity and specificity among different subclades. Comparative analysis of gene duplication events and syngenic gene pairs among different species indicated that polyploidy is beneficial for MIKC-type gene expansion. Analysis of transcriptome data within diverse tissues and stresses in *B. napus* showed tissue-specific expression of MIKC-type genes and a broad response to various abiotic stresses, particularly dehydration stress. In addition, four representative floral organ mutants (*wtl*, *feml*, *aglf-2*, and *aglf-1*) in the T0 generation were generated by editing four AGAMOUS (*BnaAG*) homoeologs in *B. napus* that enriched the floral organ variant phenotype. In brief, this study provides useful information for investigating the function of MADS-box genes and contributes to revealing the regulatory mechanisms of floral organ development in the genetic improvement of new varieties.

## Introduction

*Brassica*, a genus of Cruciferae, includes many oils, feed, and vegetable crops. *Brassica* species are the most economically valuable species providing edible oil, industrial oil, vegetables, condiments, and feed necessary for production and life (Cheng et al., 2013). Among them, the significant crops are three diploid plants: *Brassica rapa* (*B. rapa*, AA, 2n=20), *Brassica oleracea* (*B. oleracea*, CC, 2n=18), and *Brassica nigra* (*B. nigra*, BB, 2n=16), and three allotetraploid species: *Brassica napus* (*B. napus*, AACC, 2n=38), *Brassica juncea* (*B. juncea*, AABB, 2n=36), and *Brassica carinata* (*B. carinata*, BBCC, 2n=34), forming the triangle of U model. U’s triangle model, proposed by Nagaharu in 1935, is a typical theory for studying the complexity of evolutionary relationships and polyploidization among brassicas (Nagaharu and Nagaharu, 1935). With the completion and sharing of high-quality and large-scale genomic, resequencing, and comprehensive transcriptome data, a bioinformatics platform was established to explore the gene function of species in U’s triangle. *B. rapa* was the first U’s triangle species to have its reference genome (Mun et al., 2010; Wang et al., 2011; Cai et al., 2017; Zhang et al., 2018; Zhang et al., 2022), followed by *B. oleracea* (Kim et al., 2014; Liu et al., 2014; Parkin et al., 2014; Cai et al., 2020). As an important oil crop in the world, *B. napus* was sequenced in 2014 (Chalhoub et al., 2014), and many genome versions have been published (Sun et al., 2017; Song et al., 2020; Chen et al., 2021), including the winter-type oilseed rape Darmor-bzh, semi-winter oilseed rape Zhongshuang11 (ZS11), and spring-type oilseed rape Westar and so on. Subsequently, the reference genomes of *B. juncea* (Yang et al., 2016; Paritosh et al., 2021) and *B. nigra* were published successively (Paritosh et al., 2020; Perumal et al., 2020), and the reference genome of *B. carinata* was the latest to be reported (Song et al., 2021; Yim et al., 2022). To date, each of the six U’s triangle species has its reference genomes.

MADS-box genes are critical transcription factors (TFs). The term ‘MADS’ originated from four members of MADS family in fungi, plants, and animals: MCM1 in yeast, AGAMOUS in *Arabidopsis thaliana* (*A. thaliana*), DEFICIENS in snapdragon, and SERUM RESPONSE FACTOR in human (Jack, 2001). MADS-box genes are characterized by the presence of a conserved DNA-binding domain of approximately 60 amino acids in the N-terminal region of the MADS-box protein, also known as the MADS (M) domain (Yanofsky et al., 1990). MADS-box genes can be classified into Type I (M-type) and Type II (MIKC-type) subclasses according to their evolutionary relationships. Type I generally contains two exons and can further be divided into Mα, Mβ, Mγ, and Mδ subgroups based on the similarities and differences in their exon-intron features (Parenicova et al., 2003), whereas Type II typically consists of 6-8 exons and is divided into two clades, named MIKC^C^ and MIKC*. Except for the highly conserved M domain, MIKC-type TFs also comprise intervening (I), moderately conserved keratin-like (K), and variable C-terminal (C) domains (Egea-Cortines et al., 1999; Henschel et al., 2002). The M domain enables the DNA to bind CArG boxes (Riechmann and Meyerowitz, 1997; Immink et al., 2002); the I and K domains promote the formation of two or more MADS-domain proteins (Fan et al., 1997; Henschel et al., 2002; Yang and Jack, 2004), and the C domain regulates transcriptional activation of the MADS protein (Honma and Goto, 2001). To date, the functions of most MIKC*- and M-type MADS-box genes remain unclear; however, most MIKC^C^-type genes, playing vital roles in plant growth, development, and response to biotic and abiotic stress, have been elucidated (Kofuji et al., 2003).

Most TFs in the ABCDE model modulate floral organ development and belong to the MIKC-type MADS-box genes (Coen and Meyerowitz, 1991; Pinyopich et al., 2003; Zahn et al., 2006; Silva et al., 2015); examples include *FLOWERING LOCUS C* (*FLC*) (Searle et al., 2006), *SUPPRESSOR OF OVEREXPRESSION OF CONSTANS* (*SOC1*) (Lee and Lee, 2010), and *SHORT VEGETATIVE PHASE* (*SVP*) (Hartmann et al., 2000). In addition, MIKC-type genes are also involved in root (*AGL14* and *AGL21*) (Garay-Arroyo et al., 2013; Yu et al., 2014) and seed development (*SHP1/2*, *FUL*) (Ferrandiz et al., 2000; Liljegren et al., 2000). To date, the MADS-box TFs have been systematically investigated in dicotyledon plants, such as tomato (*Solanum lycopersicum* L.) (Wang et al., 2019), potato (*Solanum tuberosum* L.) (Shao et al., 2021), *B. rapa* (Duan et al., 2015), *B. oleracea* (Sheng et al., 2019), and *B. napus* (Wu et al., 2018; Zhou et al., 2022), and monocotyledon plants, such as maize (*Zea mays L.*) (Zhao et al., 2021a), wheat (*Triticum aestivum* L.) (Ma et al., 2017; Schilling et al., 2020), Foxtail Millet (*Setaria italica*) (Zhao et al., 2021b), and rice (*Oryza sativa*) (Arora et al., 2007). However, there have been no reports on the identification of MADS-box genes in three species of U’s triangle: *B. nigra*, *B. juncea*, and *B. carinata*.

In recent years, new results have enriched the functions of genes related to the ABCDE model, including *FhAG2*, a key male-specific sex determination candidate gene identified using whole-genome sequencing and assembly of two Ficus species, *F. microcarpa* (monoecious) and *F. hispida* (functionally dioecious) (Zhang et al., 2020). Owing to polyploidization and gene expansion, the copies of MIKC-type MADS-box genes increased in *B. napus* compared with that in *A. thaliana*, limiting the exploration of the function of those genes involved in floral organ development. In this study, the MADS-box gene family in the genome of U’s triangle species was identified for the first time, and gene structure characteristics, conserved domains, gene duplication, synteny events, and expression patterns of MIKC-type MADS-box genes were comprehensively analyzed. A series of *bnaag* mutants with different phenotypes were obtained using CRISPR/Cas9 technology to edit four *BnaAGAMOUS* (*BnaAG*) homologous genes in rapeseed, which offers important insights into the function of different copies of *BnaAG*.

## Materials and methods

### Sequence search and identification of MADS-box genes in species of U’s triangle

The genomic sequence, annotated file, and protein sequences of the six *Brassica* species in U’s triangle were downloaded (**Supplementary Table S1**), and the longest transcript was used for subsequent analysis. In summary, 105 AtMADS protein sequences downloaded from the TAIR database (https://www.arabidopsis.org/) (Parenicova et al., 2003) were used to perform a BLASTP search of the local protein database of the six U’s triangle species with an E-value < e^-10^ and identity ≥ 50% (Camacho et al., 2009). The hidden Markov model (HMM) profiles of SRF-TF (PF00319) and K-box (PF01486) were retrieved from the Pfam database (http://pfam-legacy.xfam.org/), and HMMER software (version 3.0) was used to search for homeodomains with an E-value < 1e^-5^. Subsequently, all MADS-box protein sequences were submitted to the National Center for Biotechnology Information Conserved Domain Database (NCBI-CDD) (https://www.ncbi.nlm.nih.gov/Structure/cdd/wrpsb.cgi) (Lu et al., 2020) and SMART database (http://smart.embl-heidelberg.de/smart/batch.pl) to examine the integrity of the MADS-box or K-box domains (Letunic and Bork, 2018). The theoretical isoelectric point (pI) and molecular weight (MW) of all candidate MADS-box proteins were predicted using ExPASy (https://www.expasy.org/) (Gasteiger et al., 2003).

### Phylogenetic tree, gene structure, and conserved motif analysis

Multiple sequence alignment of MADS-box protein sequences was performed using MAFFT software with the FFT-NS-I method (Katoh et al., 2019). Unrooted and rooted Maximum Likelihood (ML) trees were constructed using FastTree software (Price et al., 2009) and visualized using EvolView (http://www.evolgenius.info/evolview) (Zhang et al., 2012) and iTOL (http://itol.embl.de/) (Letunic and Bork, 2007). The gene structure files of all the MIKC-type genes were extracted using a genome annotation file. The conserved motifs were predicted using the MEME software, with the number of motif sets at 10 (http://meme-suite.org/tools/meme) (Bailey et al., 2009). TBtools was used to draw the exon-intron structure and conserved motif features (Chen et al., 2020).

### Gene duplication and genomic synteny analysis

The MIKC-type protein sequences of the U’s triangle species were aligned using BLASTP with an E-value < 1e^-10^ (Camacho et al., 2009). The tandem and segmental duplication events in each species were analyzed using the MCScanX program (Wang et al., 2012) and visualized using Circos (Krzywinski et al., 2009). Genomic synteny was analyzed using JCVI software and the detailed workflow reference Python version MCscan (https://github.com/tanghaibao/jcvi/wiki/).

### Expression profiles of MIKC-type MADS-box genes in *B. napus*

RNA-seq data for six tissues (roots, stems, leaves, flowers, seeds, and siliques) and four abiotic stressors (dehydration, salt, ABA, and cold) in *B. napus* were downloaded from the National Genomics Data Center (NGDC) (project ID: CRA001775) (https://ngdc.cncb.ac.cn/?lang=en). The transcriptome data of embryos and seed coats at different developmental stages and two different white powder-resistant rapeseed lines after inoculation with powdery mildew were downloaded from the NCBI database (project ID: PRJNA641876 and PRJNA778657). All transcriptome data were mapped to the Darmor-bzh genome using HISAT2 (Kim et al., 2015). The Transcripts Per Million (TPM) values were calculated using the FeatureCounts R package, and a heatmap of expression level was plotted using TBtools software (Liao et al., 2014; Chen et al., 2020).

### The sgRNA design, vector construction, and hypocotyl genetic transformation in *B. napus*

First, four *BnaAG* genes with the highest homology to *A. thaliana* (*AT4G18960*) were selected for designing single guide RNA (sgRNA). The sgRNA1 was designed to target the MADS-box and sgRNA2 to target the K-box, and then a multiplex genome editing tool CRISPR/Cas9 with two sgRNAs was used to mutate *BnaAG* genes (Xing et al., 2014). Finally, the successfully constructed recombinant plasmid (*pHSE401-BnaAG*) was transformed into *Agrobacterium tumefaciens* strain GV3101, followed by genetic transformation of 19YB437 (recipient material) using a detailed transgenic operation method described by Bhalla and Singh (Bhalla and Singh, 2008). Primers used to construct the vectors are listed in **Supplementary Table S12**.

### Identification of positive mutants and characterization of floral organ phenotype

All positive tissue culture plantlets were transplanted into humus soil and grown under a 16 h light/8 h dark photoperiod at 22°C until they reached the five-leaf stage. After four weeks of vernalization, the seedlings were transplanted into a normal environment for growth. The variant phenotypes of floral organs were observed and recorded; DNA was extracted from leaf tissues of each type of flower organ mutant, and the fragments near the two target sequences were amplified using PCR with specific primers (**Supplementary Table S12**). The PCR products and positive colonies were sequenced, and the editing events of each mutant were analyzed according to the results of Sanger sequencing.

## Results

### MADS-box genes in the six U’s triangle species

**Figure 1:**
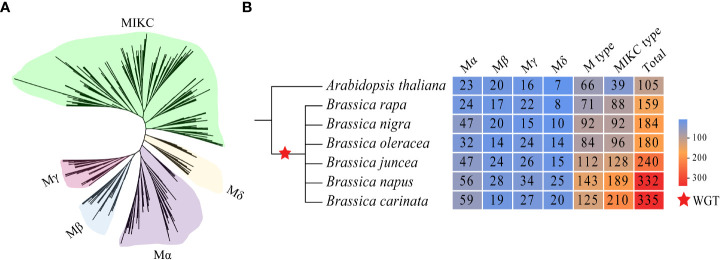
Evolutionary tree of MADS-box proteins in the six U’s triangle species and *A thaliana* and the number of subfamily members. **(A)** The phylogenetic tree of 1535 MADS proteins was constructed using the maximum likelihood method, and different colors represent different subfamilies. **(B)** The red star represents that the genome has undergone a genome-wide triplication event (WGT).

In total, 1430 MADS-box genes were identified in the genome of the six U’s triangle species. To uncover the kinship relationships of MADS-box genes among *A. thaliana* and U’s triangle species, the ML method was used to construct the phylogenetic tree based on the alignment of 105 MADSs in *A. thaliana* and 1430 MADS-box proteins in U’s triangle species (**Figure 1A**; **Supplementary Table S2**). The 1,535 MADS-box genes were divided into M-type and MIKC-type subfamilies; among them, the MIKC subfamily had the highest number of members (842), followed by the Mα (287), Mβ (143), Mγ (164), and Mδ (99) subfamilies (**Figure 1B**; **Supplementary Table S3**). The three diploid *Brassica* species *B. rapa* (AA), *B. nigra* (BB), and *B. oleracea* (CC) in the U’s triangle have undergone a whole genome triplication event (WGT) (Wang et al., 2011; Woodhouse et al., 2014) resulting in the expansion of MADS-box genes compared with *A. thaliana*; however, the gene numbers were significantly different. For example, 159, 184, and 180 MADS-box genes were identified in *B. rapa*, *B. nigra*, and *B. oleracea* genomes, respectively. In addition, although allopolyploidy facilitated the expansion of MADS-box genes, the total number of MADS-box genes identified in allotetraploid species differed from the total number of genes in its two progenitor species. For example, 240 MADS-box genes were identified in the genome of *B. juncea*, which was significantly less than the sum of genes in its progenitor species *B. rapa* (159) and *B. nigra* (184), and the number of MADS-box genes in *B. carinata* genome (335) was slightly lower than the sum of genes in its progenitor species *B. nigra* (184) and *B. oleracea* (180). The 1430 MADS-box genes were renamed based on the gene information about their distribution on chromosomes, and the protein length, molecular weight (MW), and isoelectric points (pI) of each MADS protein were analyzed synthetically to understand the basic information more comprehensively (**Supplementary Table S3**).

### Phylogenetic tree of MIKC-type MADS-box genes

**Figure 2:**
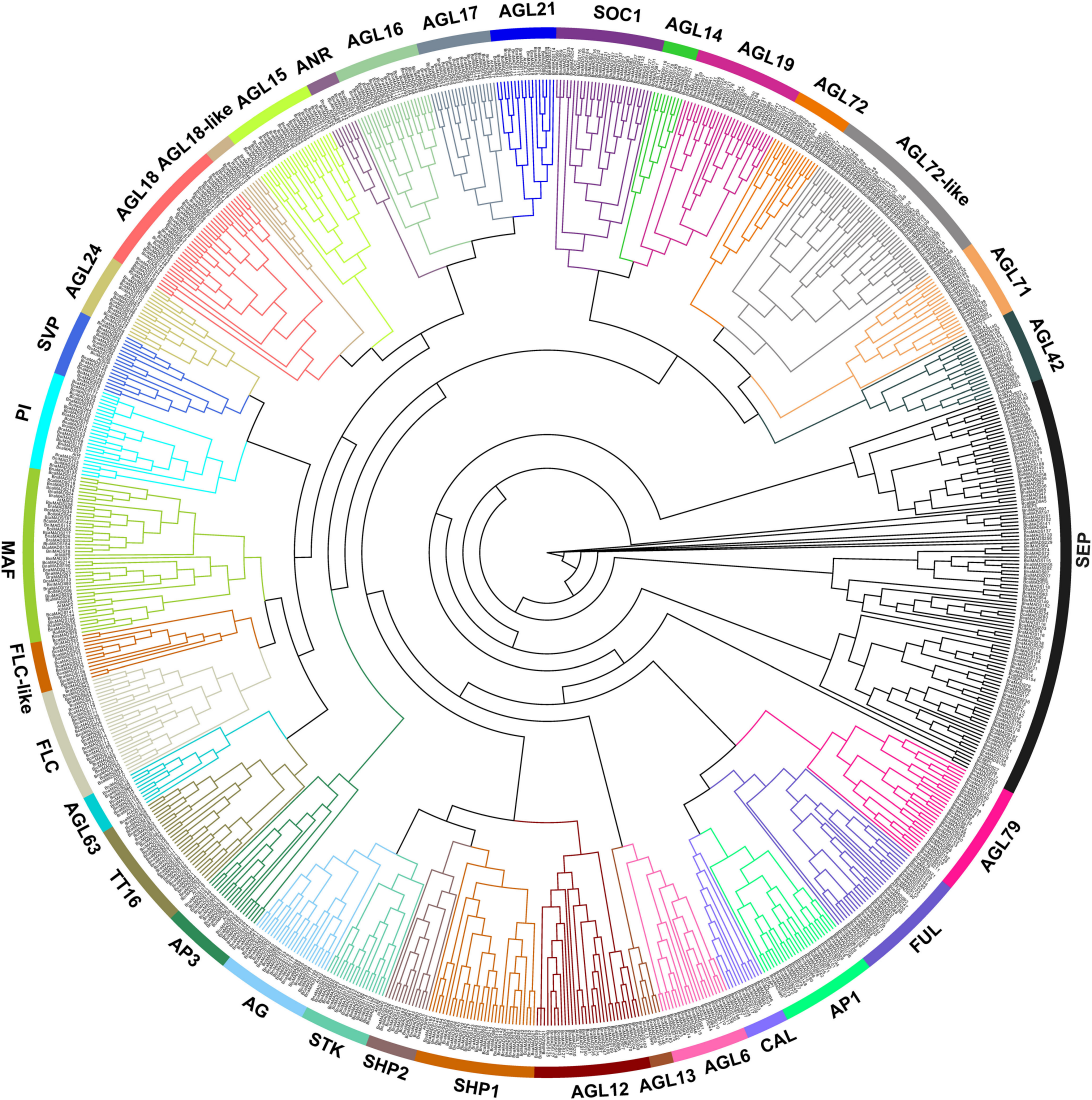
Neighbor-joining (NJ) tree of MIKC-type MADS-box genes identified in the six U’s triangle species; the genes were divided into 35 subgroups. Different colors represent different subclusters.

MIKC-type MADS-box genes are numerous, and their classification in plants is complicated. The ML phylogenetic tree was constructed for 39 MIKC-type genes in *A. thaliana* and 803 in the U’s triangle species (**Figure 2**). Subsequently, 842 MIKC-type genes were divided into 35 subclades according to classification criteria and nomenclature in *A. thaliana*. Among them, 32 subclades contained MIKC-type genes from each species, and these subclades were renamed referring to the corresponding name in *A. thaliana*. The other three subclades (AGL72-like, AGL18-like, and FLC-like) did not contain corresponding homologous genes in *A. thaliana*; therefore, their renaming was based on the name of the branch closest to their evolutionary relationships (**Figure 2**). AGL72-like subclades had the highest number of genes, which may be another sister group of AGL72 subclades. In addition, most subclades had a clear evolutionary classification, whereas the SEP subclade had the highest number of genes and a complicated branch topology, implying that SEP subclades may have undergone multiple duplication events in the U’s triangle species.

### Gene structure and conserved motifs of MIKC-type MADS-box genes

**Figure 3:**
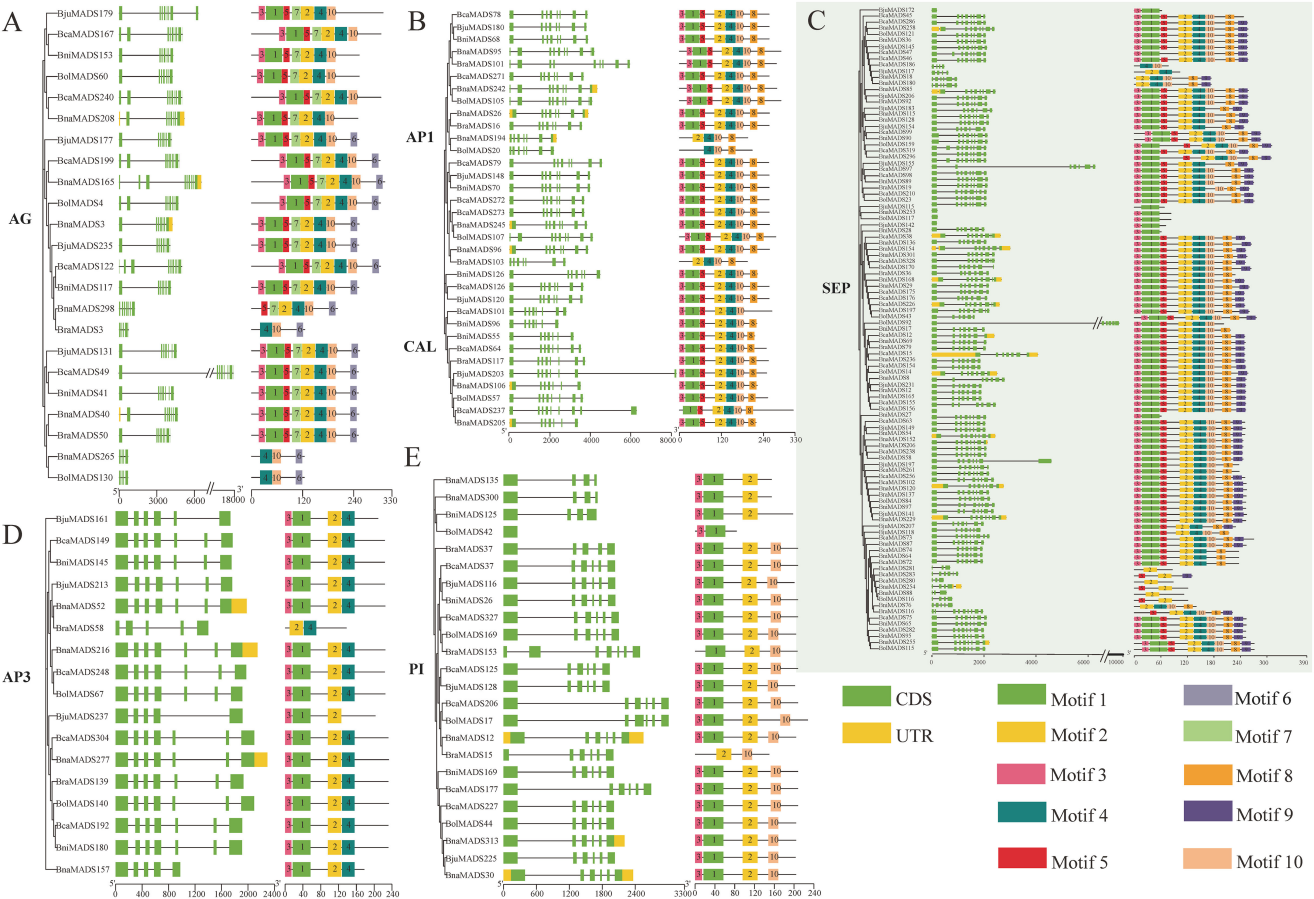
Gene structure and conserved motifs of six MIKC-type subclades [AG (**A**), AP1 and CAL (**B**), SEP (**C**), AP3 (**D**), and PI (**E**)]. The green boxes, yellow boxes, and block lines represent the exons, UTR, and introns, respectively. Ten different colored boxes represent ten distinct motifs.

The exon-intron structural characteristics of MIKC-type genes varied remarkably in different subclades; however, the number and arrangement of exon-introns were similar in the same subclade (**Figure S1**). Most of the genes in the AG and AP3 subclades comprised seven exons and six introns (**Figures 3A, D**), AP1 and CAL subclades possessed the closest evolutionary relationship, and the majority of MIKC-type genes in AP1 and CAL subgroups generally contained eight exons and seven introns (**Figure 3B**); the PI subclade (**Figure 3E**) had six exons, and the SEP subclade had eight exons (**Figure 3C**).

Ten conserved motifs were observed in 803 MIKC-type protein sequences (**Supplementary Table S4**). Motif 1 and motif 3 existed in most of the MADS-box domains, and motif 2, comparatively conserved in the K-box domain, may be responsible for the conservation of MIKC-type proteins among different subclades. However, other motifs were specifically distributed in different subclades and determined the specificity of the MIKC-type subclades (**Figure 3**; **Figure S1**). For example, comparing the conserved motifs of the AG, AP1, and CAL subclades (**Figures 3A, B**), motif 8 was only found in the AP1 and CAL subclades, whereas motifs 6 and 7 were only found in the AG subclade. Furthermore, motif 4 and motif 10 were distinctively distributed in the AP3 and PI subclades (**Figures 3D, E**), respectively, determining the specificity of these two subclades. The gene structure and conserved motifs of the other subclades are shown in **Figure S1**.

### Gene duplication and synteny of MIKC-type MADS-box genes

**Figure 4:**
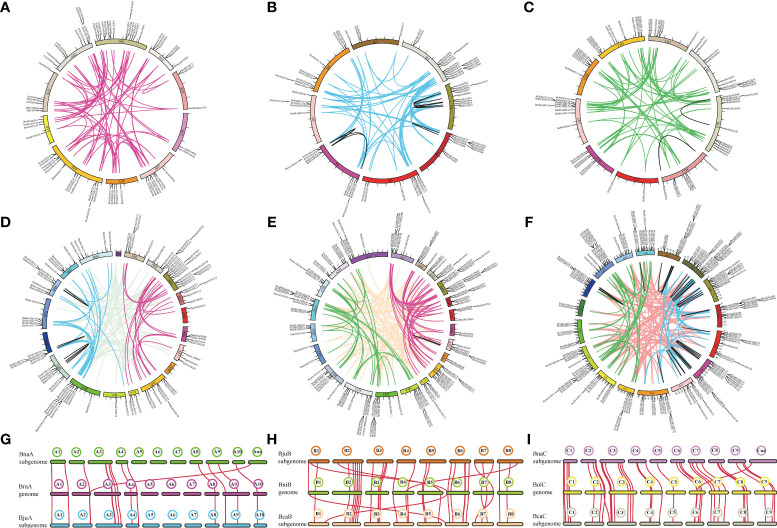
Duplication events and synteny of MIKC-type MADS-box genes. **(A-F)** Chromosome location information and segmental duplication of MIKC-type MADS-box genes. **(A–F)** indicate the segmental duplication events occurring within the genomes of *B. rapa*, *B. nigra*, *B. oleracea*, *B. juncea*, *B. napus*, and *B. carinata*, respectively. Colored lines represent replication events identified within different subgenomes, and black lines represent duplication gene pairs that occur on the same chromosome. **(G-I)** Genomic synteny between progenitor *Brassica* species *B. rapa*, *B. nigra*, and *B. oleracea*) and the allotetraploid species (*B. carinata*, *B. juncea*, and *B. napus*).

BLAST and MCScanX software were used to analyze the gene duplication events of MIKC-type genes in the U’s triangle species. A mass of tandem and segmental duplication events were detected in the genomes of *B. rapa* (3/69) (**Figure 4A**), *B. nigra* (4/75) (**Figure 4B**), *B. oleracea* (8/66) (**Figure 4C**), *B. juncea* (2/137) (**Figure 4D**), *B. napus* (4/194) (**Figure 4E**), and *B. carinata* (8/281) (**Figure 4F**). The duplication event numbers of MIKC-type genes in three diploid basic species were nearly the same. However, there was a significant difference among the three allotetraploids, particularly in segmental duplication events (**Supplementary Tables S5**, **6**), suggesting that MIKC-type genes have different evolutionary processes in the three allotetraploid species. Furthermore, the subgenome distribution of segmental duplication events in the genomes of three tetraploid *Brassica* species was analyzed. In *B. juncea*, 88 segmental duplication events occurred across the AA and BB subgenomes, 22 across the AA subgenome, and 27 across the BB subgenome (**Figure 4D**). In *B. napus*, 125 segmental duplication events occurred across the AA/CC subgenome, 41 across the AA subgenome, and 28 across the CC subgenome (**Figure 4E**). In *B. carinata*, 159 segmental duplication events occurred across the BB/CC subgenome, 85 across the BB subgenome, and 37 across the CC subgenome (**Figure 4F**). These results indicate that segmental duplication events among the three tetraploid *Brassica* species mainly occurred across the different subgenomes.

To elucidate the evolutionary process of MIKC-type genes, synteny between three allotetraploid species (*B. carinata*, *B. juncea*, and *B. napus*) and its progenitor *Brassica* species (*B. rapa*, *B. nigra*, and *B. oleracea*) was performed (**Supplementary Table S7**). A total of 15 gene pairs were identified between *B. rapa* and the A subgenome of *B. napus* and *B. juncea* (**Figure 4G**). Among them, *BnaMADS148* (*BnaAnng06990D*) may be located on chrA02 of *B. napus* because its syntenic gene pairs were all located on chrA02. In addition, 32 gene pairs were detected across the *B. nigra* genome and B subgenomes of *B. juncea* and *B. carinata* (**Figure 4H**), of which 28 gene pairs were shared on the same chromosome among the B genome of progenitor species and B subgenome of its hybridized allotetraploid species, whereas the other four were shared on different chromosomes. Additionally, 37 MIKC-type gene pairs were identified across the *B. oleracea* genome and C subgenomes of *B. napus* and *B. carinata* (**Figure 4I**). Overall, the number of syntenic gene pairs in subgenome A was significantly lower than that in subgenome B or C.

### Expression profile analysis of MIKC-type BnMADS-box genes in various tissues of *B. napus*

**Figure 5:**
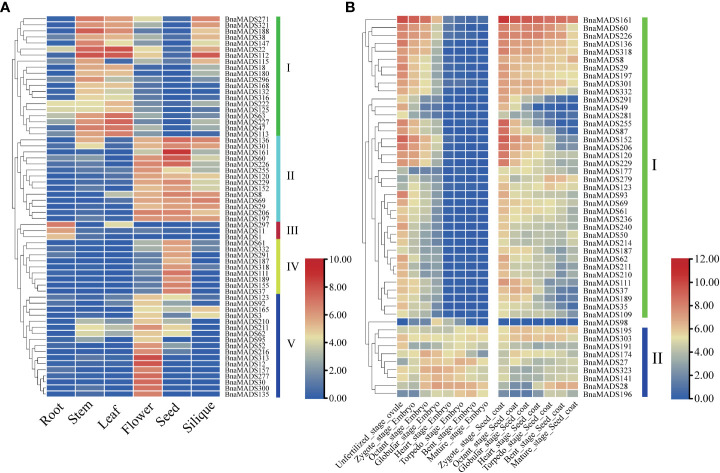
Expression patterns of MIKC-type MADS-box genes in different tissues and developmental stages. **(A)** Heatmap of TPM values in six different tissues (root, stem, leaf, flower, seed, and silique). **(B)** The gene expression trends during different unfertilized ovule, embryo, and seed coat stages.

*B. napus* is an important oil crop in the U’s triangle, and its abundant and reliable transcriptome data provide a basis for exploring the expression patterns of MIKC-type genes. Therefore, transcriptome data of various tissues, embryos, and seed coats at different developmental stages were downloaded from the public RNA-seq database to analyze the expression characteristics of MIKC-type genes. A total of 63 MIKC-type BnaMADS-box genes were screened from the expression matrix of different tissues (**Supplementary Table S8**), which were classified into five subgroups according to their expression patterns (**Figure 5A**). Subgroup I genes were highly expressed in stems and leaves, and some genes were also highly expressed in siliques and other tissues; however, all genes were expressed at low levels in the seed. Subgroup II genes were mainly expressed in flowers, seeds, and siliques, but their expression was low in roots. The subgroup III genes were root-specific. Nine genes, including especially *BnaMADS37*, *BnaMADS111*, *BnaMADS155*, and *BnaMADS189*, in subgroup IV were highly expressed in seeds. The genes in subgroup V, including *BnaMADS12*, *BnaMADS30*, *BnaMADS300*, and *BnaMADS313*, were only highly expressed in the flowers. Overall, the expression patterns of the 63 BnMADS-box genes were tissue-specific, suggesting that these genes may have vital biological functions in tissues during specific developmental processes.

To explore the expression patterns of MIKC-type BnaMADS-box genes at different developmental stages of the embryo and seed coat, the expression patterns of 48 BnaMADS-box genes were divided into two major subgroups (**Figure 5B**; **Supplementary Table S9**). Subgroup I genes were mainly expressed during the unfertilized ovule stage and first three periods of embryonic development (zygote, octant, and globular stages), whereas subgroup II genes were generally expressed during the embryo and seed coat development stages, especially in the middle and late stages of embryo development, such as *BnaMADS27* was highly expressed in torpedo and bent stage embryos, *BnaMADS141* and *BnaMADS32*3 were highly expressed in octant, globular, and heart stage embryos. In addition, *BnaMADS98* was highly expressed in globular stage embryo, whereas it was less expressed during seed coat development. Therefore, analyzing the expression pattern of MIKC-type genes in *B. napus* provided a guide for exploring the biological functions of MIKC-type genes in various tissues during the entire developmental process.

### Expression profiles of MIKC-type BnMADS-box genes under biotic and abiotic stressors in *B. napus*

**Figure 6:**
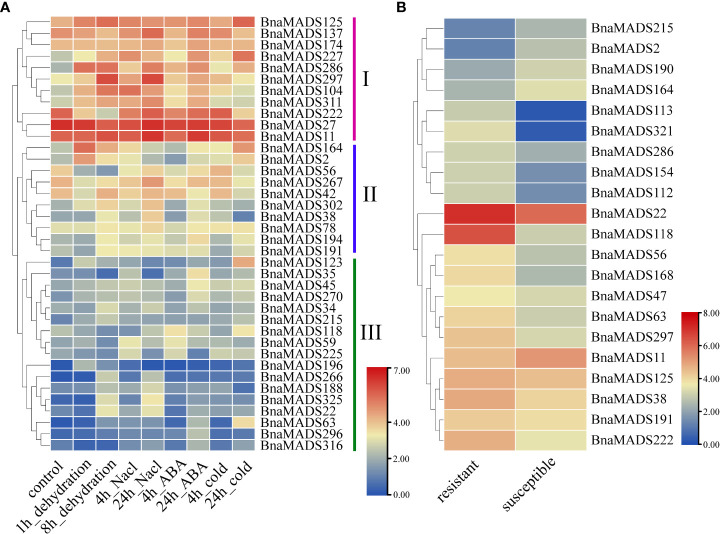
Expression of MIKC-type MADS-box genes in response to biotic and abiotic stresses. **(A)** Expression profiles of genes in rapeseed leaves under four different abiotic stressors (dehydration, salt, ABA, and cold). **(B)** The leaves expression patterns of two different white powder-resistant rapeseed lines after inoculation with powdery mildew.

To determine the mechanism of MIKC-type genes responding to biotic and abiotic stress, transcriptome data for dehydration, salt, ABA, and cold stress were downloaded from the public RNA-seq database (**Supplementary Table S10**). Based on gene expression trends under abiotic stress, the 38 MIKC-type genes were divided into three categories: subgroups I, II, and III (**Figure 6A**). Subgroup I genes were expressed under various types of abiotic stresses. *BnaMADS227*, *BnaMADS286*, and *BnaMADS297* were significantly upregulated after dehydration, salt, ABA, and cold treatment, whereas *BnaMADS104* and *BnaMADS311* were more susceptible to dehydration, salt, and ABA stress. *BnaMADS222* was downregulated at 1 h and 8 h of dehydration stress, indicating that *BnaMADS222* was more sensitive to dehydration stress. A small subset of the genes in subgroup II was sensitive to abiotic stress. *BnaMADS2* and *BnaMADS164* were induced by dehydration and cold treatment; *BnaMADS38* and *BnaMADS302* were upregulated under dehydration and salt stress, and *BnaMADS56* was significantly downregulated after dehydration stress. Subgroup III genes had low expression under abiotic treatments; however, individual genes were upregulated after stress treatment. For example, *BnaMADS123* and *BnaMADS63* were significantly highly expressed after 24 h of cold treatment, and *BnaMADS35* was upregulated after 24 h of ABA treatment. The rapeseed leaf transcriptome data of resistant and susceptible lines after inoculation with powdery mildew were downloaded for further analysis (**Figure 6B**; **Supplementary Table S11**). A total of 21 MIKC-type genes responded to powdery mildew stress, of which 16 genes, including *BnaMADS113*, *BnaMADS118*, and *BnaMADS321*, showed significantly higher expression in resistant strains than in sensitive strains. Furthermore, *BnaMADS2*, *BnaMADS11*, *BnaMADS164*, *BnaMADS190*, and *BnaMADS215* showed higher expression in susceptible strains than in resistant strains.

### Sequence analysis of *BnaAG* and vector construction

**Figure 7:**
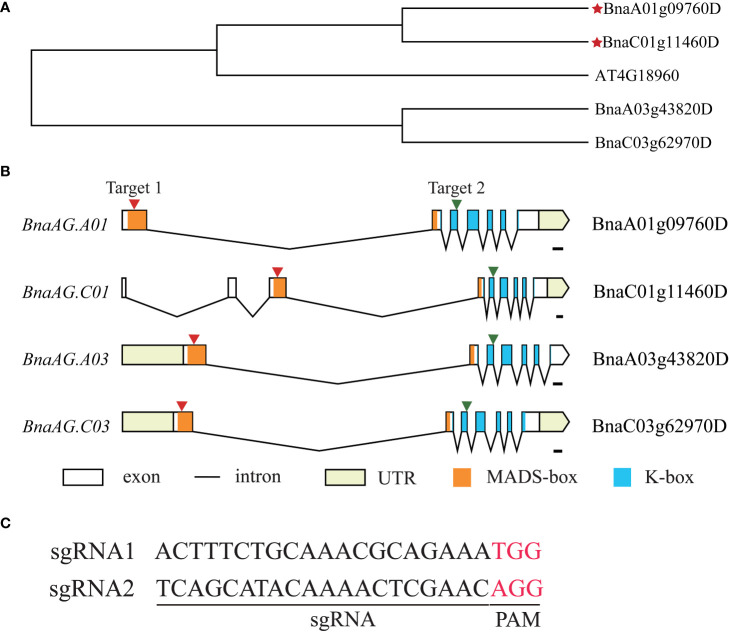
Construction of vectors for targeting *BnaAG* homologous genes. **(A)** NJ tree of *BnaAG* homologous genes in *B napus* and *A thaliana*. The red stars represent the *BnaAG* homologs of *B napus* closely related to the *AG* gene in *A thaliana*. **(B)** Gene structure, conserved domain, and distribution of sgRNA1 and sgRNA2 targeting four homologous genes of *BnaAG*. The black line represents introns, and the black boxes represent the exon. The red arrows indicate the location of sgRNA targets on the exon, and the orange and blue boxes represent MADS-box and K-box domains, respectively. **(C)** Sequence information of two sgRNA, PAM is indicated in red.

To further verify the contribution of *BnaAG* genes to floral organ development, four highly homologous *BnaAG*s, *BnaA01g09760D*, *BnaA03g43820D*, *BnaC01g11460D*, *BnaC03g62970D* (also named *BnaAG.A01*, *BnaAG.A03*, *BnaAG.C01*, and *BnaAG.C03*, respectively), located on chromosomes A01, A03, C01, and C03, respectively, were isolated by Blastp with *AtAG*. Phylogenetic tree analysis showed that *BnaAG.A01* and *BnaAG.C01* had the closest relationships with *AtAG* (**Figure 7A**). The observation of gene structure features showed that most of the *BnaAG* genes contained seven exons, whereas *BnaAG.C01* was composed of nine exons but had no effect on its conserved domain at the gene structure level (**Figures 7B**). Based on multiple alignments of *BnaAG* homologous genes, CRISPR/Cas9 vectors were constructed to target the conserved regions of four *BnaAG* homoeologs. In brief, the consensus sequence on the first exon of *BnaAG.A01*, *BnaAG.A03*, *BnaAG.C03*, and the third exon of *BnaAG.C01* were targeted by sgRNA1. SgRNA2 targeted the consensus sequence on the third exon of *BnaAG.A01*, *BnaAG.A03*, *BnaAG.C03*, and the fifth exon of *BnaAG.C01* (**Figures 7C**). Based on these results, sgRNA1 was located on the MADS-box domain and sgRNA2 on the K-box domain.

### CRISPR/Cas9 mediated mutation in *BnaAG* homoeologs and enriched floral organ mutant phenotypes

**Figure 8:**
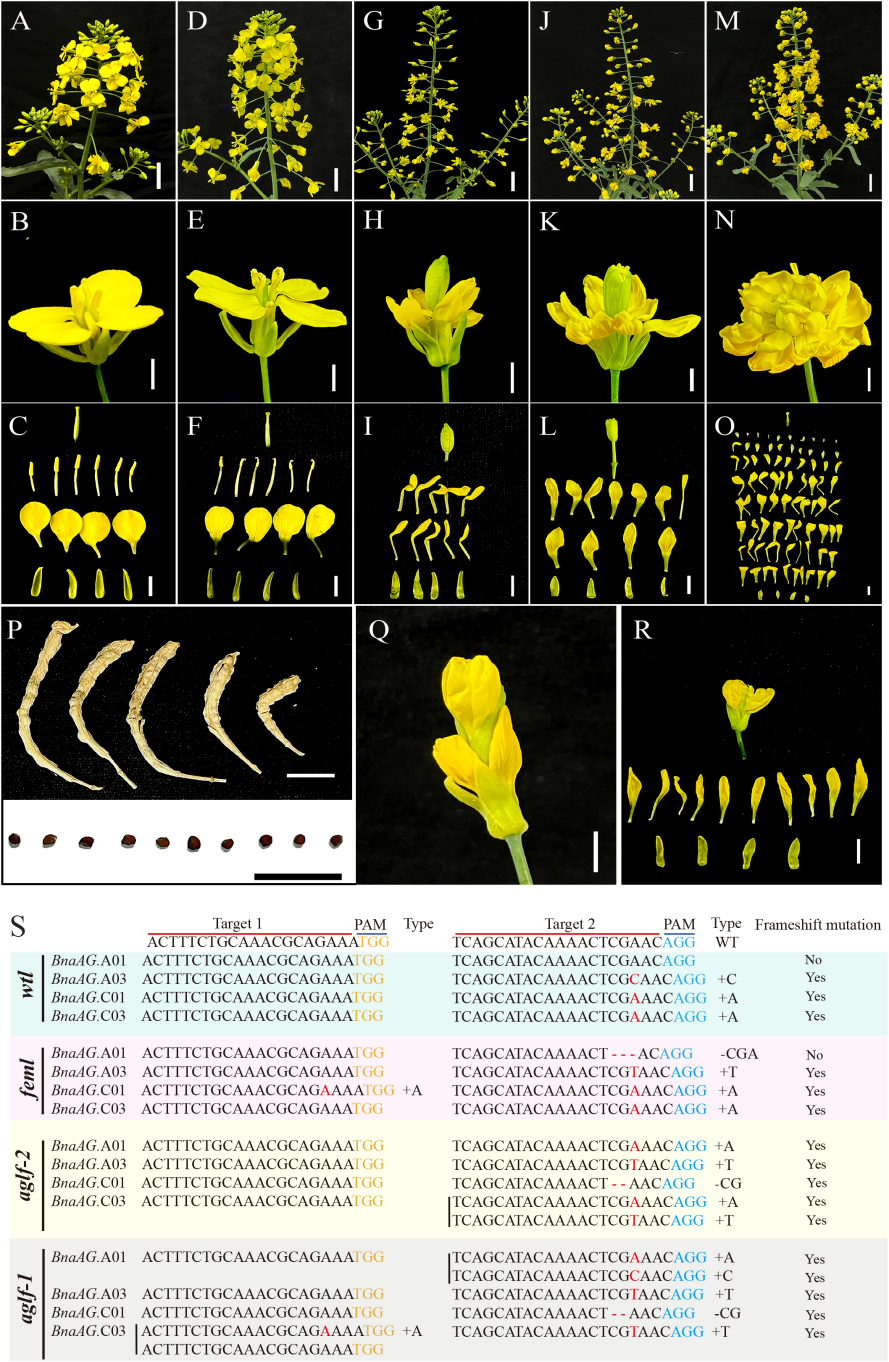
Phenotype and target sequence validation of the T0 generation of the gene-edited *BnaAG* genes. mInflorescences of WT **(A)**, *wtl* **(D)**, *feml* **(G)**, *aglf-2* **(J)**, and *aglf-1* **(M)**. Scale bars, 3 cm. The flowers of WT **(B)**, *wtl* **(E)**, *feml* **(H)***, aglf-2* **(K)**, and *aglf-1* **(N)** plants. Scale bars, 0.5 cm. Components from the anatomizing flowers of WT **(C)**, *wtl* **(F)**, *feml* **(I)**, *aglf-2* **(L)**, and *aglf-1* **(O)**. Scale bars, 0.5 cm. **(P)** Mature siliques and seeds of *feml* lines after cross-pollination. Scale bars, 1 cm. **(Q, R)** Flower **(Q)** and components from the anatomizing flower **(R)** of *aglf-2* during the flowering stage. **(S)** Validation of target sequences for various mutant phenotypes. Yellow and blue fonts represent the PAM of target sequences 1 and 2, respectively. Red ‘-’ means deletions, and red font represents single base insertions. Yes or no indicates that the *BnaAG* gene underwent a frameshift mutation.

After the genetic transformation of the rapeseed hypocotyl mediated by *Agrobacterium tumefaciens*, more than 40 T0 generation hygromycin B-positive plants were obtained, of which 20 lines consisted of four concentric wheels and showed no difference from the wild-type (WT), whereas the other lines showed a disorganized floral structure. Therefore, T0 generation lines can be classified into four types based on their floral organ mutation phenotypes. To verify the mutation type, plants of all phenotypes were screened using Sanger sequencing, and the editing efficiency of sgRNA1 was found to be significantly lower than that of sgRNA2 (**Figures S2**, **3**). Specifically, compared to wild type (WT) (**Figures 8A–C**), there were no phenotypic differences found in *wt-like* (*wtl*) (**Figures 8D–F**). Sequencing results showed that no editing events existed in target 1, and all *BnaAG* homologs had a single base inserted except *BnaAG.A01* in target 2 (**Figure 8S**). The *female flower-like* (*feml*) mutant (**Figures 8G–I**), which led to the conversion of stamens into petals and expansion of carpels, generated low seed-setting and distorted siliques after cross-pollination (**Figure 8P**). The sequencing results showed that no editing events happened except for single base insertion in *BnaAG.C01* of target 1. *BnaAG.C01*, *BnaAG.C03*, and *BnaAG.A03* had a single base insertion in target 2, whereas *BnaAG.A01* had a deletion of three bases (CGA) in target 2 that did not cause frameshift mutation (**Figure S4**). The next mutant, like *ag-2* mutant in *A. thaliana*, gave rise to the following phenotype: six stamens replaced by six petals, and the carpel replaced by a new flower with an elongated pedicel, in which stamens and carpels were replaced by petals (also called *agamous-like flower-2* (*aglf-2*) (Yanofsky et al., 1990; Tsutsui and Higashiyama, 2017) (**Figures 8J–L, Q, R**). Target 1 of the *BnAG* homologous gene was not edited; however, insertion or deletion occurred in all *BnAG* homologous genes in target 2, resulting in a frameshift mutation. The phenotype of the last mutant was similar to the *ag-1* mutant in *A. thaliana* (Yanofsky et al., 1990); its outermost whorl had four normal sepals, while its third whorl stamens and fourth whorl carpel were all replaced by a multitude of petals. The resulting flower mutant, *agamous-like flower-1* (*aglf-1*), contained more than 85 petals (**Figures 8M–O**), in which *BnaAG.A01, BnaAG.C01, and BnaAG.A03* with base insertion or deletion in target 2 caused a frameshift mutation, whereas *BnaAG.C03* had a single base insertion in both the target squences, resulting in a frameshift mutation.

## Discussion

The U’s triangle model represents the six globally important *Brassica* species and explains the genetic relationships among them. The *B. carinata* reference genome was published in 2021, marking the start of functional genomics. It also promoted the exploration of genetic evolution and molecular design breeding in the U’s triangle species (Song et al., 2021; Yim et al., 2022). The MADS-box gene family is an important transcription factor that functions in floral homeostasis, morphogenesis, angiosperm reproductive development, and vegetative development (Okada and Shimura, 1994; Becker et al., 2000; Alvarez-Buylla et al., 2019).

### The MIKC-type MADS-box genes were significantly expanded in the genome of six U’s triangle species

Previous studies showed that a total of 160, 91, and 307 MADS-box genes have been identified in *B. rapa*, *B. oleracea*, and *B. napus* genomes, respectively. However, there was no related research regarding MADS-box genes in *B. nigra*, *B. juncea*, and *B. carinata* genomes (Duan et al., 2015; Wu et al., 2018; Sheng et al., 2019). With the disclosure of the genome information of the remaining three *Brassica* species (*B. nigra*, *B. juncea*, and *B. carinata*), this study was the first to perform the systematic identification and classification of MADS-box genes in U’s triangle species. A total of 159 MADS-box genes were identified in the *B. rapa* genome, which is consistent with previous results (Duan et al., 2015). However, 180 MADS-box genes were investigated in the *B. oleracea* genome, which was approximately twice the number previously reported (Sheng et al., 2019), and 332 MADS-box genes were identified in the *B. napus* genome, which was 25 more than originally reported (Wu et al., 2018) (**Figure 1B**). The differences in the number of members may be confined to the reference genome quality of previously assembled *Brassica* species. The MADS-box genes can be divided into M-type and MIKC-type according to their phylogenetic relationships. The gene number ratio of the two types was 1.69 (66:39) in *A. thaliana*, 0.8 (71:88) in *B rapa*, 1.0 (92:92) in *B. nigra*, 0.87 (84:96) in *B. oleracea*, 0.87 (112:128) in *B. juncea*, 0.75 (143:189) in *B. napus*, and 0.59 (125:210) in *B. carinata*, indicating that the ratio of M-type and MIKC-type genes decreased gradually compared with that of *A. thaliana*. Moreover, the genome of the three diploid U’s triangle species has undergone genome-wide triploid events during the evolutionary process (Wang et al., 2011; Woodhouse et al., 2014), leading to a significantly higher ratio of MIKC-type genes in *A. thaliana* and three diploid species (*A. thaliana*:*B. rapa*:*B. nigra*:*B. oleracea* = 39:88:92:96) than that of M-type genes (*A. thaliana*:*B. rapa*:*B. nigra*:*B. oleracea* = 66:71:92:84). The function of M-type genes in plants is poorly understood, whereas MIKC-type genes are implicated in plant growth and development and environmental adaptation (Kofuji et al., 2003; Yang et al., 2016; Guo et al., 2021). Therefore, the significant expansion of MIKC-type genes in the U’s triangle species may be an adaptive evolution.

### The tissue-specific expression patterns of MIKC-type BnaMADS-box genes

Public transcriptome data helps effectively identify valuable genes. In this study, a large number of tissue-specific MIKC-type BnaMADS-box genes were discovered in various tissues and during embryo development processes in *B napus*, of which *BnaMADS1* and *BnaMADS297* were highly expressed in roots (**Figure 5A**). *AtAGL14* (*XAL2*, *AT4G11880*), a homologous gene of *BnaMADS297* in *A. thaliana*, which binds to the CArG box (a cis-regulatory element of *PIN1* and *PIN4)*, regulates root elongation by upregulating the abundance of PIN protein (Garay-Arroyo et al., 2013). *AGL21* (*AT4G37940*), a homolog of *BnaMADS1*, mainly regulates lateral root development, especially during lateral root growth under low-nitrogen conditions (Yu et al., 2014). *BnaMADS111* is specifically expressed in seeds and highly expressed during the early stage of seed coat development (zygote, octant, and globular stage) (**Figure 5B**); its homolog *AGL32* (*TT16*, *AT5G23260*) modulates the development of the sub-epidermal integument cell layer in *A. thaliana* seeds (Coen et al., 2017). *BnMADS12* and *BnMADS313* were specifically highly expressed only in flowers, and their homologous gene *PI* in *A. thaliana* acts as a floral homeotic gene mainly involved in the regulation of petal and stamen development (Goto and Meyerowitz, 1994). *BnaMADS28* is highly expressed during the later stages of embryo and seed coat development, and its homologous gene *AGL15* (*AT5G13790*) facilitates somatic embryogenesis in *A. thalian*a (Paul et al., 2022). *BnaMADS161* is highly expressed during the early stage of embryo development and throughout the seed coat development process, and its homologous gene *AGL11* (*STK*, *AT4G09960*) in *Arabidopsis* is involved in seed and silique development (Di Marzo et al., 2022).

### MIKC-type BnaMADS-box genes are widely involved in drought stress in *B. napus*

Plant responses to biotic and abiotic stresses reflect resilience to external challenges. Plants usually respond to drought stress by flowering early to complete their life cycle, a phenomenon called drought escape (Waadt et al., 2022). Transcriptome data analysis showed that some BnaMADS-box genes involved in flowering time are also highly expressed under drought stress, possibly regulating drought escape. For example, *AGL20* (*SOC1*, *AT2G45660*), a homologous gene of *BnaMADS227* in *A. thaliana*, is upregulated by *ABF3* and *ABF4* under drought stress, thus promoting flowering (Hwang et al., 2019). *BnaMADS227* is not only upregulated under drought stress but also induced by salt, ABA, and cold stress, indicating that *BnaMADS227* participates in drought response and other abiotic stresses. In addition, *BnaMADS2* and *BnaMADS164* also respond to various abiotic stresses, such as dehydration and cold stress, and homologous *AGL16* (*AT3G57230*) negatively regulates drought stress through stomatal density and movement in *A. thaliana* (Zhao et al., 2020). Thus, we speculated that these two genes may be involved in drought or cold stress. The expression of some genes, such as *BnaMADS118*, *BnaMADS113*, and *BnaMADS321*, was high in resistant strains and low in susceptible strains; however, the homologs of these genes in *Arabidopsis* are not reportedly involved in biotic stress. Exploring the mechanism of these genes in powdery disease can enrich the function of MIKC-type BnaMADS-box genes.

### The *BnaAG* homolog genes may be functionally redundant

Polyploid plants enriched the floral organ phenotype due to the complex gene copy number but interfered with the systematic study of floral organ development. *AG* is responsible for stamen identity, carpel identity, and floral meristem determination (Theissen et al., 2000; Dreni and Kater, 2014). In this study, CRISPR/Cas9 technology was used to knock-out four *BnaAG* genes, and four lines with significant phenotypic differences in the T0 generation were obtained: *wtl*, *feml*, *aglf-2*, and *aglf-1* (**Figure 8**; **Figure S3**). The *BnaAG.A01* was not edited in *wtl*, suggesting that the presence of only one copy of *BnaAG* can maintain the normal development of floral organs. However, the amino acid mutation of *BnaAG.A01* did not cause a termination mutation, and the frameshift mutation occurred on the other three homologous genes, resulting in the *feml* phenotype. In addition, termination mutations of four *BnaAG* homologs in *aglf-2* and *aglf-1* resulted in phenotypes similar to those in *Arabidopsis* (Yanofsky et al., 1990). These results confirmed that *BnaAG* genes were involved in the development of third and fourth whorls in flowers, suggesting that there may be functional redundancy among the four *BnaAG* genes.

### *BnaAG* may be a sex-identification marker gene in *B. napus*

*AG* is responsible for the floral organ sex determination of plants (Zhang et al., 2020; Wang et al., 2021). The flowers of *B. napus* are monoecious, and the creation and application of male sterile materials in rapeseed can greatly enrich the utilization of heterosis. The *feml* phenotype, similar to the female flower, was found in the T0 generation after editing *BnaAG.* Although the carpel was deformed, a few seeds could be obtained by cross-pollination, implying that the *feml* was a female flower without stamens. Thus, *BnaAG* may be a sex-determining marker gene in *B. napus.*

## Conclusions

In conclusion, 1430 MADS-box genes were identified in the six U’s triangle species and classified into M-type and MIKC-type based on their evolutionary relationships. Gene structure, conserved motifs, duplication events, and synteny between the subgenomes of different MIKC-type genes were analyzed. The expression profiles of different tissues revealed that the MIKC-type genes exhibited tissue-specific expression. Transcriptome analysis of genes induced by abiotic and biotic stress showed that MIKC-type MADS-box genes responded to external stimuli. Moreover, four types of floral organ mutants were created by editing *BnaAG* homologous genes, which revealed the mechanisms by which *BnaAG* regulates floral organ development in rapeseed. Our study provides novel insights into the regulation of floral organ development in *Brassica* species and may help develop new genetic varieties.

## Data availability statement

The datasets presented in this study can be found in online repositories. The names of the repository/repositories and accession number(s) can be found in the article/**Supplementary material**.

## Author contributions

SWH designed the experiments. MS wrote the manuscript. MS and YFZ executed the experiments. QJ and SHH analyzed the data. RA and NC prepared the figures. JM and YTZ revised the manuscript. All authors contributed to the article and approved the submitted version.

## Supplementary material

Supplementary Figure 1 Gene structure and conserved motifs of MIKC-type subclades except for AG, AP1, CAL, AP3, PI, and SEP subclades.

Supplementary Figure 2 Editing efficiency of sgRNA-1 and sgRNA-2.

Supplementary Figure 3 Multiple sequence alignment of BnaAG.A01 in WT and *feml*.
