# Supplementary figures and images for: Systematic analysis of MADS-box gene family in the U’s triangle species and targeted mutagenesis of BnaAG homologs to explore its role in floral organ identity in Brassica napus
Source: Front Plant Sci. 2023 Jan 12;13:1115513. doi: 10.3389/fpls.2022.1115513 (PMC9878456; doi:10.3389/fpls.2022.1115513)

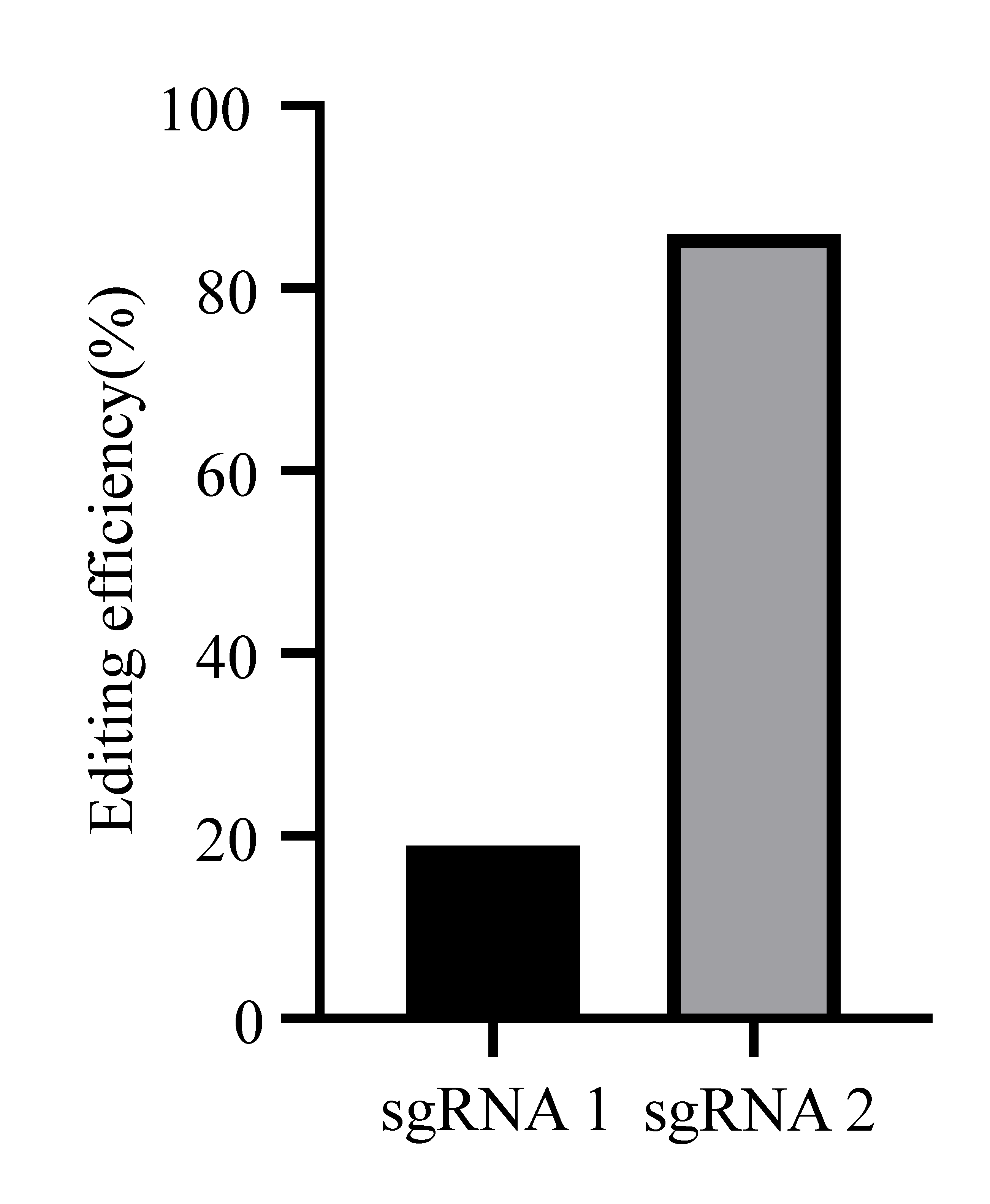

Supplement: Supplementary Figure 1 — Gene structure and conserved motifs of MIKC-type subclades except for AG, AP1, CAL, AP3, PI, and SEP subclades. [file Image_1.tiff]

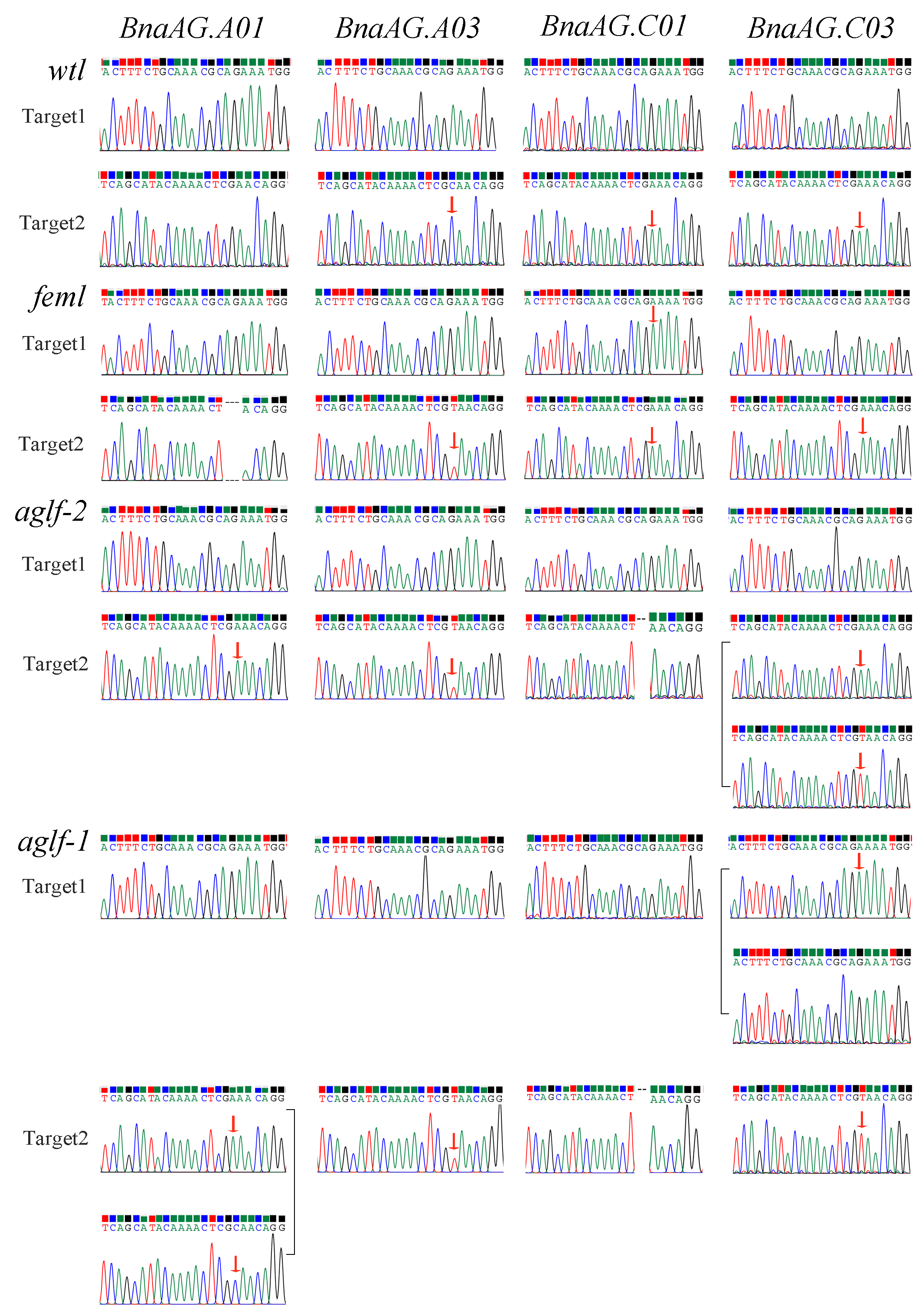

Supplement: Supplementary Figure 2 — Editing efficiency of sgRNA-1 and sgRNA-2. [file Image_2.tiff]

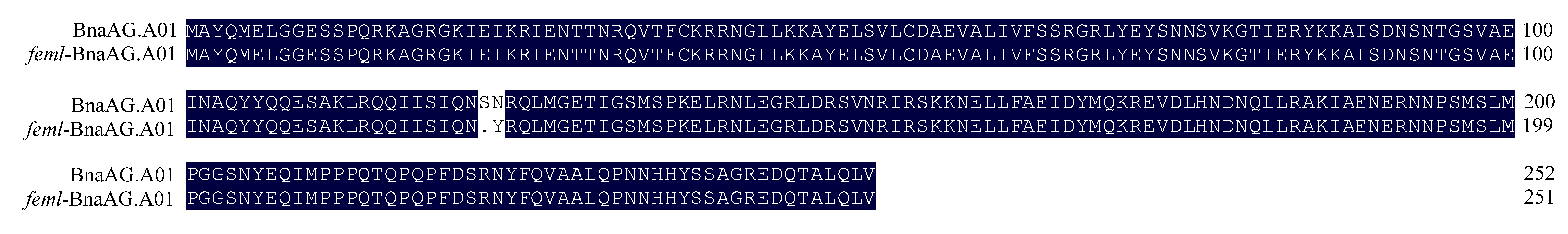

Supplement: Supplementary Figure 3 — Multiple sequence alignment of BnaAG.A01 in WT and feml. [file Image_3.tiff]

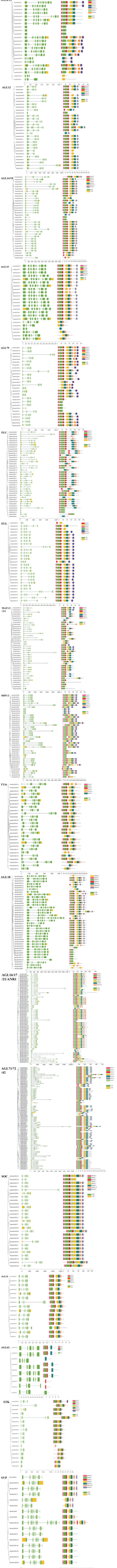

Supplement: Supplementary file 4 [file DataSheet_1.pdf]
